# Predicting the future risk and outcomes of severe heart failure and coronary artery disease with machine learning in the UK Biobank Cohort

**DOI:** 10.1371/journal.pone.0329461

**Published:** 2025-09-10

**Authors:** Karim Taha, Heather J. Ross, Mohammad Peikari, Brigitte Mueller, Chun-Po S. Fan, Edgar Crowdy, Yas Moayedi, Filio Billia, Cedric Manlhiot

**Affiliations:** 1 Department of Medicine, The Red Rogers Centre for Heart Research, Peter Munk Cardiac Centre, University Health Network, University of Toronto, Toronto, Ontario, Canada; 2 Department of Pediatrics, The Blalock-Taussig-Thomas Pediatric and Congenital Heart Center, Johns Hopkins School of Medicine, Johns Hopkins University, Baltimore, Maryland, United States of America; Universita degli Studi di Roma Tor Vergata, ITALY

## Abstract

**Background:**

In order to seriously impact the global burden of heart failure (HF) and coronary artery disease (CAD), identifying at-risk individuals as early as possible is vital. Risk calculator tools in wide clinical use today are informed by traditional statistical methods that have historically yielded only modest prediction accuracy.

**Methods:**

This study uses machine learning algorithms to generate predictions models for the development and progression of severe HF and CAD. Participants (~485,000 followed in the UK Biobank over 7 years) were stratified by cardiac status at the time of enrollment (asymptomatic, high-risk and affected); separate prediction models were built for each stratum. Participants were split between a training set (80%) and holdout dataset (20%), all performance metrics are reported for the holdout dataset.

**Results:**

Out of 6 machine learning algorithms screened, artificial neural networks (ANN) most successfully predicted future disease across the various strata (area under the curve: 0.77–0.86 for 10/12 models), results were very consistent between methodologies. Models trained using ANN showed excellent calibration in all strata and across the entire spectrum of risk (0.4–1.2% average observed/predicted difference across 10 deciles of risk). Key predictive features included age, frailty, adiposity, history of hypertension and diabetes, tobacco use and family history of heart disease and were consistent between models for HF and CAD.

**Conclusions:**

When deployed as a patient-facing application, the prediction models presented here will be able to provide both user-specific predictions and simulate the effect of changes in lifestyle and of prophylaxis interventions, thus resulting in an individualized patient counselling and management tool.

## Introduction

Cardiovascular disease (CVD) is the most common non-communicable disease globally, responsible for an estimated 17.8 million deaths in 2017 [1]. By the year 2030, the UN Sustainable Development Goals effort aims to reduce premature mortality from non-communicable diseases by a third [2]. Heart failure and coronary artery disease (CAD) are progressive diseases wherein the majority of disease progression occurs prior to the development of symptoms and disease progression occurs over a number of years. In order to seriously impact the global burden of this disease, identifying at-risk individuals as early as possible and prior to symptom development is of paramount importance. It is equally important to identify the presence of risk factors that increase risk of progression or death in symptomatic or already diagnosed patients. Knowing this may allow changes via lifestyle and risk factor modification earlier in the natural history of these disease processes [3].

Current guidelines to prevent coronary artery disease and heart failure (HF) focus on primary prevention based on predicted risk from widely used calculators, and primarily inform the initiation of statins in high-risk asymptomatic patients. The recommended risk calculator varies regionally, with the American College of Cardiology/American Heart Association and Canadian Cardiovascular Society recommending the Framingham Risk Score (FRS) [4–6], while in the UK, the National Institute for Health and Care Excellence (NICE) recommends the QRISK2 score [7]. These risk prediction tools are typically built using multivariate regression models that combine a small number of well-established risk factors. They historically have had limited predictive performance (AUC low 0.70s or below), which becomes particularly evident for subsets of the population like the < 30 age group and asymptomatic women in the case of FRS [8,9].

Machine learning (ML) is an increasingly important analytical tool that can improve risk prediction accuracy by using data-driven feature selection, including undescribed patterns and data and by considering complex interaction between variables. Though the use of machine learning is growing, there are still only a handful of large dataset-driven studies that employ a variety of well-established ML methods for the study of cardiovascular disease and the prediction of outcomes [10,11]. The majority are limited by dataset size, depth of features included, and/or number of ML methods compared against one another to reach adequate performance metrics [12,13].

Some of the most commonly used ML methods in the literature are: linear support vector machines (SVM) (a linear classifier), random forest (a tree-based ensemble method), neural networks (a deep learning method), AdaBoost and gradient boosting machines (boosting ensemble methods). Many of these methods have been used both as comparators against one another, and individually against CSMs to evaluate effectiveness.

The aim of this study is thus to use and compare against one another a variety of currently widely used ML methods to develop an accurate prediction model for the development of HF and CAD. This study uses the UK biobank and applies the models against three distinct cohorts of patients: disease-free asymptomatic adults at baseline, adults at high risk of developing CVD, and lastly, progression of disease in those who are already diagnosed.

## Methods

### Study design

The UK Biobank (UKB) [14] is the world’s largest national health resource aimed at improving treatment, diagnosis, and prevention of serious and life-threatening diseases. The initiative has successfully to date recruited over 500,000 people aged between 40–69 years during years 2006–2010 across the UK (England, Wales and Scotland). The UK Biobank project has approval from the North West Multi-Centre Research Ethics Committee (MREC) and the Community Health Index Advisory Group (CHIAG). Recruitment was done centrally via coordinated identification and invitation from population-based registers (such as those held by the National Health Service (NHS)) of potentially eligible people living within a reasonable traveling distance of an assessment centre. Hence, it ensures that the participant population is a random but representative cross-section of the UK population. Prior to participation, all participants completed the consent procedures using the direct data entry system into a touch-screen interface. Participant assessment at enrollment included a review of their previous medical history, lifestyle and exposure questionnaires, physical assessment and blood sampling. Longitudinal follow-up data (hospitalizations and deaths) was collected on an ongoing basis after enrollment, both from the primary care record and the NHS registry, for as long as participants still consented. Secondary use of the UK Biobank data for this specific study was reviewed and approved by the Research Ethics Board at University Health Network.

### Participant stratification

Participants in this study were divided into 3 strata based on their cardiac status at the time of enrollment. The rationale for our division of the population into three strata is grounded in the clinical decision-making of practitioners day to day while treating patients. There is a large variance of risk across these three cohorts of patients. A patient who has already suffered a myocardial infarction is treated very differently than one who has not had an index event. Further, the patient with typical risk factors but who has not had an index event, also receives a different approach from their clinician. Thus, assessing each cohort in isolation offers a higher granularity of features that may affect patients seen clinically on the far end of the spectrum of risk.

The first stratum included participants with existing heart disease defined as self-reported but physician-confirmed diagnosis of any of the following conditions: heart/cardiac disease, heart attack/myocardial infarction, arrhythmia, valve disease, cardiomyopathy or heart failure.

The second stratum included high-risk participants defined as patients who were symptomatic but not given a formal diagnosis of CAD or HF by a medical practitioner at any point prior to data collection. These participants reported exertional angina, chest pain, orthopnea, and/or other heart failure-type or CAD-type symptoms. These items were selected based on expert review and experience from the cardiologist and clinical lead on the team after thorough evaluation of all symptoms available in the intake questionnaire. This stratum also included participants taking medications that are usually prescribed to patients with cardiac pathology or with pathologies significantly increasing the risk of future pathologies. Thus, any participant taking anti-anginal, diuretic, angiotensin converting enzyme inhibitor (ACEI), angiotensin receptor blocker (ARB), beta-blocker and calcium channel blocker medications were included in the high-risk stratum.

The third and last stratum included all other participants not meeting the criteria for either of the first 2 strata. This was considered the low-risk stratum of patients who have not had an index event yet.

### Risk factor assessment at baseline

Collection of lifestyle, exposures and health-related information was done through self-completed questionnaires and interviews with medical professionals, which complemented a series of physical measurements and biological samples also collected at the baseline assessment. The emphasis in the baseline questionnaire was to concentrate on known and potential risk factors for outcomes that are already, or are projected to become, important public health concerns for the adult population. The touch-screen self-administered questionnaire was then used to collect the majority of information. The questionnaire can be categorized into the following broad topic areas of interest: socio-demographics and occupation; lifestyle exposures (including smoking, alcohol, physical activity and diet); early life exposures; psychological state; cognitive function; family history of illness; and medical history and general health). The inclusion and exclusion of baseline physical measurements at the assessment for the UKB were considered with respect to relevance, reliability and resources. Baseline physical measurements were also collected in the UKB at the time of questionnaire completion and included: blood pressure, pulse rate, weight, height, waist and hip circumference and hand grip strength. As part of the baseline assessment, biological samples were collected and blood biochemistry was measured [15,16]. The complete list of predictive features used in prediction models is provided in S1 Table.

Genome-wide genotype data for all participants were generated using the Affymetrix UK BiLEVE Axiom array (initial 50,000 participants) and the Affymetrix UK Biobank Axiom Array (remaining 450,000 participants), along with an imputed dataset of over 90 million single nucleotide polymorphisms (SNP) [17]. From these arrays, we pre-selected 61 SNPs in 25 genes that were previously identified as being high relevance to HF and CAD [18–25]. Specific criteria for inclusion of SNPs were genetic response to a drug active on circulatory system receptors, a SNP with known association to development or accelerated progression of HF/CAD, or a SNP with proximity to a genetic locus with bearing on CVD (S2 Table).

### Feature pre-selection

We selected appropriate baseline risk factors for inclusion into the model. These were selected as any variable that is either known to have a clinical bearing on CAD and HF, or may potentially play a role, but is not yet described or understood in today’s standard of practice. These included, but were not limited to, age at recruitment, sex, sociodemographic variables including combined household income, education, ethnicity, physical activity, sleep habits, smoking, diet by short- and long-term recall, alcohol intake, drug use, early life factors such as family history and health/medical history (**Table 1**). Physical medical attributes including height, weight, blood pressure, pulse rate, bone densitometry, and spirometry were included as well. A comprehensive set of bloodwork and urine data was included, including biochemistry, hematological data and urinalysis. All above features served as the baseline inputs into the machine learning models in this study. We deliberately separated the cohort into males and females because a number of risk factors apply only to one group or the other (such as hormone replacement therapy). This ensured that the ML models we employed could properly use sex-specific data.

**Table 1:** Prevalence of outcomes stratified by sex and risk groups.

	Males		Females	
	# participants	# outcomes	# participants	# outcomes
Coronary artery disease				
Low-risk	145,084	10,829 (7.5%)	196,821	6,036 (3.1%)
High-risk	44,018	3,388 (7.7%)	44,327	1,381 (3.1%)
Affected	21,834	2,595 (11.9%)	12,300	684 (5.6%)
Heart failure				
Low-risk	143,700	2,903 (2.0%)	196,259	2,058 (1.0%)
High-risk	43,596	925 (2.1%)	44,255	483 (1.1%)
Affected	20,097	1,260 (6.3%)	11,866	362 (3.1%)

### Outcomes assessment

All enrolled participants provided explicit permission at enrollment for investigators to access their medical and other health-related records in the future. Access to such records provided accurate follow-up information related to cause-specific mortality and other health events. Several sources and systems were used by the UK Biobank staff to ascertain cause of death, disease occurrence and other health-related information among participants during long-term follow-up. These sources include long-standing NHS information technology systems, the Scottish Morbidity Record, and the National Card Record Service. The full list is extensive and detailed online in the UK Biobank protocol online resource [26].

The UK Biobank recruited and collected baseline assessments on intake between years 2007 and 2010 and all participants are continually followed through their medical and death register records indefinitely as long as the patient still consents. For the purpose of this study, we ascertained outcomes 7 years after enrollment. This threshold was selected empirically to minimize the number of patients that would be excluded because of insufficient follow-up. Therefore, labels for the ML modeling are created by binary encoding outcomes in the span of 7 years after patient enrollment. The primary outcomes of interest in this study were hospitalization with a new diagnosis of HF and CAD (in the low and high-risk strata only) or death from HF or CAD (including both primary and secondary causes of death). Hospital admissions and mortality associated with a diagnosis of ischemic heart disease or myocardial infarction (ICD codes and subcodes between I.21 and I.25) met the criteria for the CAD outcome while ICD codes related to heart failure (ICD code I.50 and subcodes) met the criteria for the HF outcome.

### Analytic approach, data preparation

Categorical fields were converted to binary data points using one-hot-encoding methods. Relevant fields were combined to create one data field. Therefore, fields with less than 5% frequency were combined with other closest and relevant fields. All rows and columns with more than 20% missing data (if any) were removed from the analysis. Missing values of binary features were replaced by 0 and those that correspond to continuous variables were imputed using the R’s Multivariate Imputation by Chained Equations (MICE) library. After processing, a total of 315 variables were available for prediction models.

### Modelling approach

Data was divided into two mutually exclusive subsets of 70 and 30 percent (train and test sets) respectively. A five-fold cross-validation scheme and grid search for hyperparameter tuning were used for training and internal validation on the training set. After tuning, the final model was built using the best-performing parameter set. The final model was then applied to the later holdout subset (test set) to evaluate the performance of each learning model and their performance metrics. Both subsets of data were the same for all learning methods. To compare the performance of the classifications, the area under the receiver operating curve (ROAUC), sensitivity, specificity, positive and negative predictive values (PPV, NPV), positive and negative predictive rates (PPR, NPR), and F-1 scores were calculated separately in the training dataset and in the holdout dataset. An F-1 score is a harmonic mean of PPV and sensitivity that represents the accuracy of a classifier. Confidence intervals for performance metrics in holdout sets were derived through bootstrapping (1000 resamples, 20%−80% random sample for each iteration). Calibration curves were used to evaluate the closeness of distributions of class probabilities to that of the expected class labels. In a well-calibrated model, the class probabilities should reflect the true likelihood of the actual class label.

To address class imbalance during training, we considered two strategies. The first involved assigning higher weights to the minority class while keeping the majority class weight at one, ensuring the model placed greater emphasis on learning from the underrepresented cases. The second approach utilized Synthetic Minority Over-Sampling Technique (SMOTE) to generate synthetic samples for the minority class. After evaluating both methods, we found that class weighting provided the best overall performance and was used in the final models. This also served to calibrate model probability and adjust the prediction threshold. We trained a logistic regression (LR) model using the logarithmically transformed training probabilities and the actual labels. This allowed us to obtain the coefficients and intercept of the LR model. To calibrate the probabilities in the test set, we applied the LR model’s intercept and coefficients to the test probabilities. Subsequently, we selected a new threshold in a manner that maintained the same proportion of positive and negative cases as in the training cohort, using the newly calibrated probabilities. Heatmaps with hierarchical clustering were generated for all study strata and outcomes to allow for the examination of the profiles for each group. Variable importance for each model was summarized using a ridge regression meta-model. A ridge regression model was trained using the calibrated test set probability values (as the target value) and the test data. The regression coefficients were averaged over all models for a given outcome and ranked according to their sign and magnitude to reflect the most and least important variables. As such, large coefficients (either positive or negative) were the most important variables and coefficients close to 0 were the least important variables [27].

### Algorithm selection

We applied 6 different ML methods to each stratified dataset (a total of 36 models) in order to examine the performance of a variety of learning methods and to identify the strengths and weaknesses of each method across the cohorts. The 6 binary classifiers were: artificial neural networks (ANN), gradient boosting method (GBM), random forest (RF), bootstrap logistic regression (LR) with stepwise feature selection, Lasso logistic regression, and support vector machines (SVM). A brief overview of the methods for each of these models is provided in the associated appendix. Python 3.7.7 and R 3.5.3 were used for all analyses.

## Results

A total of 485,833 participants were included in this study, 217,404 males (44%) and 268,429 females (56%). Over 7 years of follow-up (2010–2017), 15,532 coronary event outcomes and 3,762 heart failure outcome events over 3,400,831 patient-years were recorded. Distribution of participants by sex and risk strata along with the prevalence of outcomes for each group is provided in **Table 1**. Prevalence of outcomes for both the low and high-risk groups was low and similar to each other; while the prevalence of outcomes was much higher in participants already affected at baseline. Prevalence of outcomes were higher in male than female participants.

**Fig 1:**
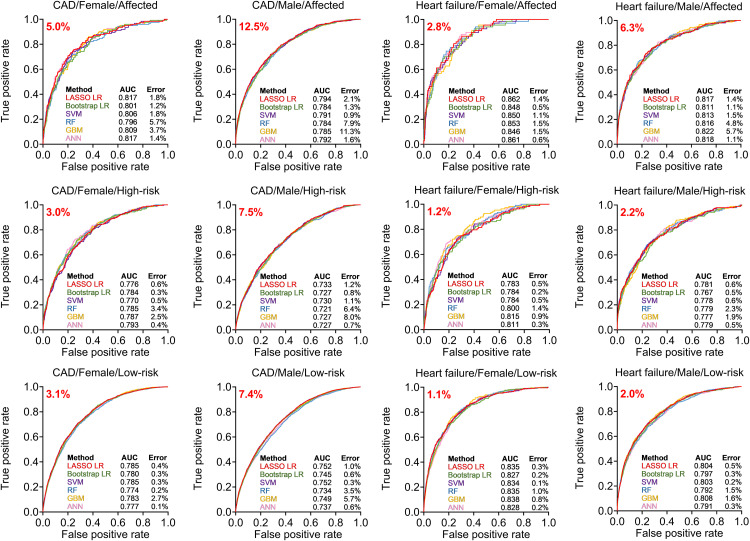
AUC and average calibration error plots in the hold-out. Comparison of AUC and average calibration error (average absolute observed vs. predicted difference over 10 deciles of risk) in the hold-out dataset for all methods tested for this study for each of the outcomes/strata combinations. Number is red represents outcome prevalence. Abbreviations: ANN, artificial neural network; AUC, area under the curve; CAD, coronary artery disease; GBM, gradient boosted machines; LR, logistic regression; RF, random forest; SVM, support vector machine.

Model test AUC was fairly consistent (±2%) between the various methods (Fig 1). However, the calibration errors were disparate between methods, with GBM and RF showing substantially higher errors than the other 4 methods (Fig 1), particularly at the high end of the risk spectrum. While the other 4 model building strategies had comparable performance, the models based on the ANN strategy showed the best combination of high AUC and low calibration error and was the most consistent across all models. Thus, we elected to use the ANN models as the primary modeling strategy for all strata and outcomes.

Performance metrics for our ANN models in each stratum for both the training and holdout datasets are reported in **Table 2**. Individual ANNs for all subsets of participants had an AUC in the holdout dataset between 0.77 and 0.86 with the exception of the model for CAD in low- and high-risk males with an AUCs in the low 0.70’s consistent with all other models for these strata. Prediction models for either outcome showed high specificity but poor sensitivity, a classic feature with rare outcomes. Model sensitivity improved in the affected groups and was substantially higher for CAD than it was for heart failure. Finally, while performance declined in the validation set vs. the training set, that decline was limited, thus alleviating concerns of overfitting.

**Table 2:** Performance metrics in the holdout dataset for prediction models created using the artificial neural network strategy in each risk/sex strata.

	AUC	Sensitivity/Precision	Specificity	NPV	FPR	FNR	FDR	Accuracy	F1 score
CAD									
Female									
Affected	0.82 (0.76–0.87)	0.29 (0.18–0.42)	0.96 (0.95–0.97)	0.96 (0.95–0.97)	0.04 (0.03–0.05)	0.71 (0.58–0.82)	0.71 (0.57–0.82)	0.93 (0.91–0.94)	0.29 (0.18–0.40)
High-risk	0.79 (0.75–0.83)	0.18 (0.11–0.25)	0.97 (0.97–0.98)	0.97 (0.97–0.98)	0.03 (0.02–0.03)	0.82 (0.75–0.89)	0.82 (0.75–0.89)	0.95 (0.94–0.96)	0.18 (0.11–0.24)
Low-risk	0.78 (0.76–0.80)	0.16 (0.13–0.19)	0.97 (0.97–0.98)	0.97 (0.97–0.98)	0.03 (0.02–0.03)	0.84 (0.81–0.87)	0.84 (0.80–0.87)	0.95 (0.94–0.95)	0.16 (0.13–0.19)
Male									
Affected	0.79 (0.76–0.82)	0.41 (0.35–0.49)	0.92 (0.90–0.93)	0.92 (0.90–0.93)	0.08 (0.07–0.10)	0.59 (0.51–0.65)	0.59 (0.52–0.65)	0.85 (0.84–0.87)	0.41 (0.36–0.47)
High-risk	0.73 (0.70–0.75)	0.25 (0.20–0.30)	0.94 (0.93–0.95)	0.94 (0.93–0.95)	0.06 (0.05–0.07)	0.75 (0.70–0.80)	0.75 (0.70–0.80)	0.89 (0.88–0.90)	0.25 (0.20–0.29)
Low-risk	0.74 (0.72–0.75)	0.25 (0.23–0.28)	0.94 (0.94–0.94)	0.94 (0.94–0.94)	0.06 (0.06–0.06)	0.75 (0.72–0.77)	0.75 (0.72–0.77)	0.89 (0.88–0.89)	0.25 (0.23–0.28)
Heart failure									
Female									
Affected	0.86 (0.80–0.92)	0.28 (0.13–0.45)	0.98 (0.97–0.99)	0.98 (0.97–0.99)	0.02 (0.01–0.03)	0.72 (0.55–0.88)	0.72 (0.54–0.88)	0.96 (0.95–0.97)	0.28 (0.13–0.44)
High-risk	0.81 (0.75–0.87)	0.10 (0.02–0.21)	0.99 (0.99–0.99)	0.99 (0.99–0.99)	0.01 (0.01–0.01)	0.9 (0.79–0.98)	0.90 (0.81–0.98)	0.98 (0.97–0.98)	0.10 (0.02–0.19)
Low-risk	0.83 (0.80–0.85)	0.13 (0.08–0.18)	0.99 (0.99–0.99)	0.99 (0.99–0.99)	0.01 (0.01–0.01)	0.87 (0.82–0.92)	0.87 (0.82–0.91)	0.98 (0.98–0.98)	0.13 (0.09–0.18)
Male									
Affected	0.82 (0.77–0.86)	0.36 (0.27–0.45)	0.96 (0.95–0.97)	0.96 (0.95–0.97)	0.04 (0.03–0.05)	0.64 (0.55–0.73)	0.64 (0.55–0.73)	0.92 (0.91–0.93)	0.36 (0.28–0.44)
High-risk	0.78 (0.72–0.83)	0.18 (0.09–0.28)	0.98 (0.98–0.99)	0.98 (0.98–0.99)	0.02 (0.01–0.02)	0.82 (0.72–0.91)	0.82 (0.73–0.91)	0.96 (0.96–0.97)	0.18 (0.09–0.27)
Low-risk	0.79 (0.76–0.82)	0.18 (0.13–0.23)	0.98 (0.98–0.99)	0.98 (0.98–0.99)	0.02 (0.01–0.02)	0.82 (0.77–0.87)	0.82 (0.77–0.87)	0.97 (0.96–0.97)	0.18 (0.13–0.23)

Metrics in parentheses are 95% confidence interval derived from 1,000 bootstraped resamples. Abbreviations: CAD, coronary artery disease; FDR, false discovery rate; FNR, false negative rate; FPR; false positive rate; NPV, negative predictive value.

**Fig 2:**
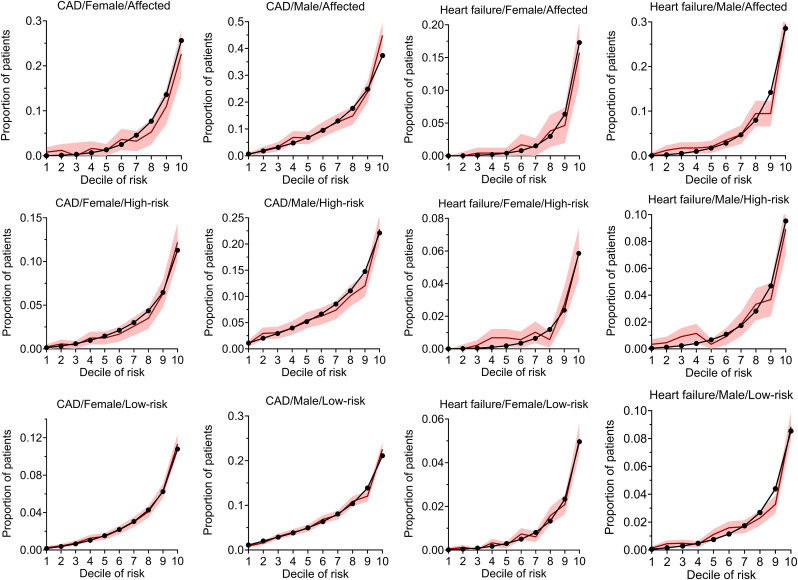
Calibration metrics in the hold-out dataset for the prediction models. Calibration metrics in the holdout dataset for the prediction models created using the artificial neural network strategy in each risk/sex strata. The observed prevalence of outcomes in each decile is depicted with their 95% confidence intervals in red bar while the predicted prevalence is depicted as a black line/circle. Abbreviation: CAD, coronary artery disease.

The calibration curves for the ANN models in each stratum are provided in Fig 2. The degree of model calibration was generally high for all models with an average difference incidence between predicted and observe prevalence per decile of risk of 0.4% for the low and high-risk groups and 1.2% for the 2 affected strata. Amongst the different strata of risk we defined, the ANN model performed best in the low-risk stratum, particularly at higher deciles of risk, and performed equally for both males and females. Models for CAD tend to be better calibrated than those for HF.

**Fig 3:**
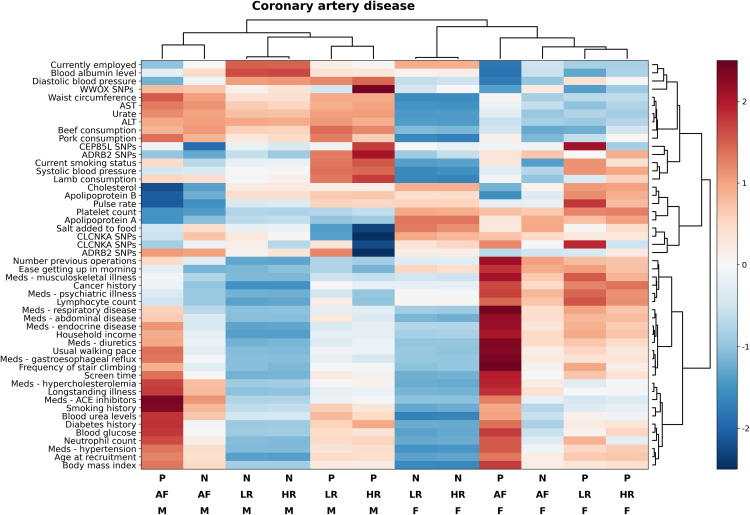
Heatmap of patient risk profiles for CAD stratified by risk strata and outcomes. Heatmap (with hierarchical clustering) of patient risk factor profile for coronary artery disease stratified by risk strata and outcomes. Only factors that are either differentially expressed between groups and outcomes or those that substantially contributed to the final prediction models are reported, some highly correlated risk factors are not shown to improve readability. Abbreviations: ACE, angiotensin-converting enzyme; ADRB2, adrenoreceptor beta 2; AF, affected; ALT, alanine transaminase; AST aspartate aminotransferase; CEP, centrosomal protein; CLCNKA, chloride channel protein CLC-Ka; HR, high-risk; LR, low-risk; Meds, medication; N, negative; P positive; SNP, single nucleotide polymorphism; WWOX, WW domain containing oxidoreductase. (Bottom legend: P = positive, N = negative, F = female, M = male, LR = low risk, HR = high risk, AF = affected).

**Fig 4:**
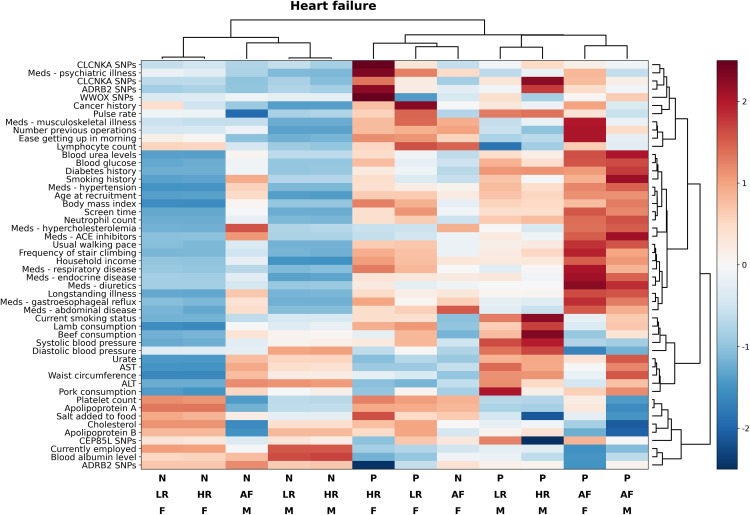
Heatmap of patient risk profiles for HF stratified by risk strata and outcomes. Heatmap (with hierarchical clustering) of patient risk factor profile for heart failure stratified by risk strata and outcomes. Only factors that are either differentially expressed between groups and outcomes or those that substantially contributed to the final prediction models are reported, some highly correlated risk factors are not shown to improve readability. Abbreviations: ACE, angiotensin-converting enzyme; ADRB2, adrenoreceptor beta 2; AF, affected; ALT, alanine transaminase; AST aspartate aminotransferase; CEP, centrosomal protein; CLCNKA, chloride channel protein CLC-Ka; HR, high-risk; LR, low-risk; Meds, medication; N, negative; P positive; SNP, single nucleotide polymorphism; WWOX, WW domain containing oxidoreductase. (Bottom legend: P = positive, N = negative, F = female, M = male, LR = low risk, HR = high risk, AF = affected).

Figs 3 and 4 show substantial differences in overall risk profile for CAD and HF between the different risk and sex strata. Differences in risk profiles were seen for CAD vs. HF, between participants with heart disease at baseline vs. low and high-risk patients and between patients who developed CAD and HF vs. those who didn't. The only strata that showed some consistency in risk profile were the low and high-risk groups and this was consistent for both CAD and HF and for male and female participants. At the time of baseline assessment, in low and high-risk participants, both HF and CAD were associated with older age, higher body mass index, hypertension, diabetes, hypercholesterolemia, as well as elevated glucose, neutrophil and white blood cell count and urea. Endocrine, gastrointestinal reflux or respiratory disease, previous history of cancer and number of previous operations were all associated with elevated odds of HF and CAD in all strata. Elevated liver enzymes were strongly associated with developing heart failure in female participants but not in male patients; while higher pulse rate was associated with developing heart failure in all subgroups but was not associated with the risk of developing CAD.

In terms of lifestyle, a lower degree of physical activity, higher screen time, increased consumption of red meat, amount of salt added to food and tobacco smoking were all associated with increased risk of developing both CAD and HF. Finally, gene polymorphisms in the *ADRB2, CPE85L, CLCNKA* and *WWOX* genes were associated with developing HF in high-risk participants. Factors associated with adverse outcomes in participants that were affected at baseline were generally similar to unaffected low and high-risk participants with some differences in intensity. The effect of pulse rate, liver enzymes, and cholesterol control was magnified in affected participants, while nutrition (other than salt intake), employment status and genetics (other than *ADRB2* polymorphisms were reduced in this group.

**Fig 5:**
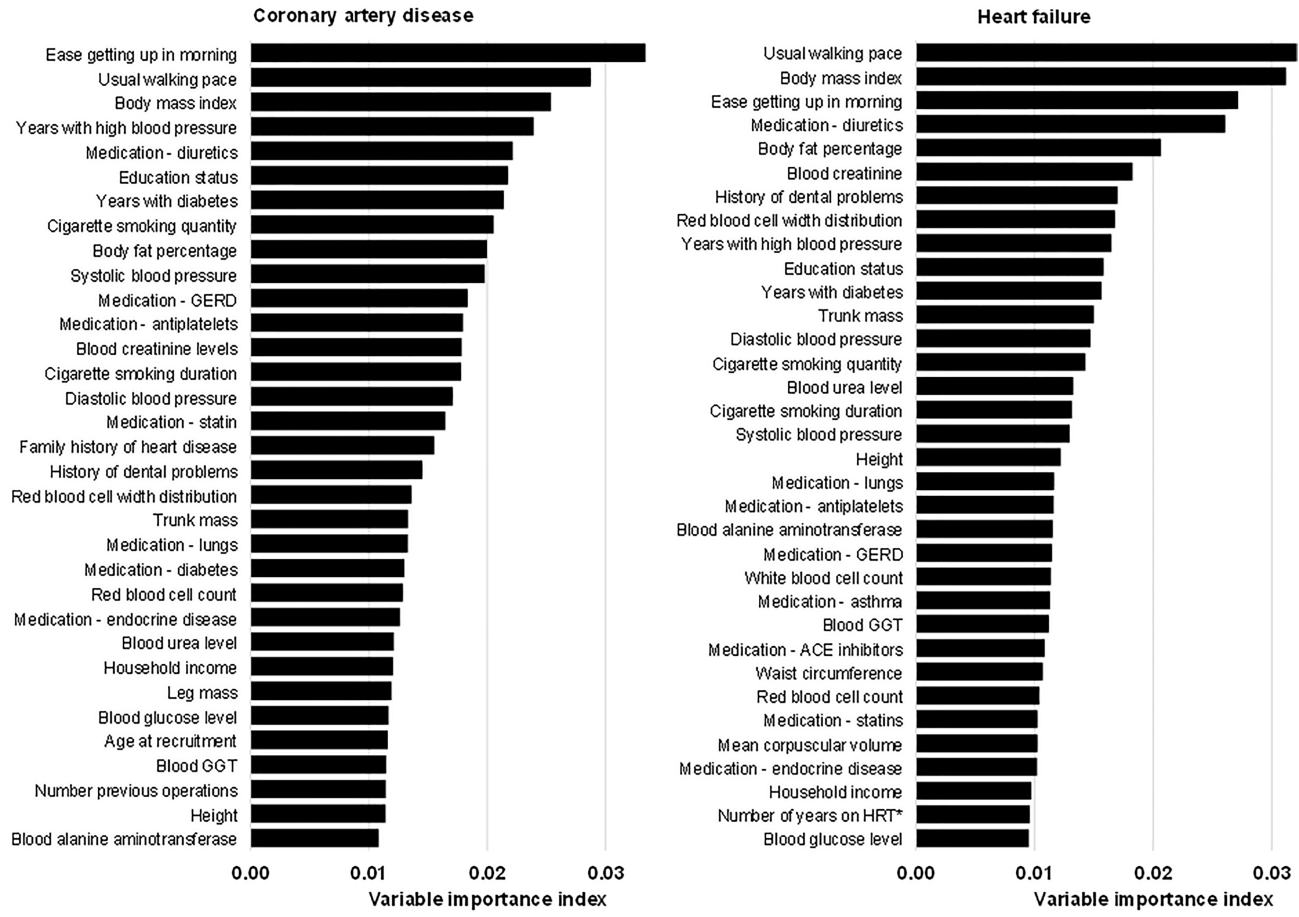
Feature importance plot averaged over all prediction models from ANN modeling strategy. Feature importance averaged over all prediction models created using the artificial neural network strategy. Only features with an importance index above the 90^th^ percentile are reported. Features with an asterisk are relevant only to a single sex. Abbreviations: GERD, gastroesophageal reflux disease; GGT, gamma-glutamyl transferase.

Average variable importance indices for both HF and CAD (across all strata) are reported in Fig 5. There was considerable overlap between the list of influential features for both HF and CAD. Overall age, frailty, adiposity and previous medical history (hypertension and diabetes) had the highest variable importance index. Lifestyle exposures, particularly smoking and salt and meat intake were also among the variables with a high variable importance index. Importantly, other lifestyle and genetic factors, while different between the various strata, did not contribute substantially to the predictive models.

## Discussion

Using the UK Biobank to develop prediction models for the development of HF and CAD, we found that ANN models yielded reasonably high AUC-ROC, on par with widely used clinical risk stratification tools like the Framingham Risk Score and the QRISKII model (8), with excellent model calibration, even for low-risk patients. This suggests that while predicting long-term events in specific patients might remain challenging given the stochastic nature of cardiovascular events, our models can be used for accurate risk stratification. Our models are made to mirror clinical workflows where patients who have important risk factors (such as hypertension, diabetes, and high BMI) are intuitively considered and managed differently. Our analysis revealed important distinction in risk factors for events between risk categories and sex and highlights that healthy lifestyle is critical in low risk patients while management of secondary risk factors should be the focus for high-risk patients.

Our aim in this study is to build on existing algorithms that have now become second nature to the practicing clinician’s risk assessment. Building on existing CSMs such as the Framingham Risk tool will advance the field of risk profiling more robustly than attempting to replace highly validated clinical tools considered the gold standard. Risk profile development for each stratum yielded a host of variables that often coincided with the current best knowledge of CAD and HF natural history. This spanned across biomarkers, lifestyle, and genetic components.

The Framingham model, despite its wide use, has limitations, typically overestimating risk in low-risk patients and underestimating risk in high-risk patients [28]. There is of course no shortage of other risk calculators, and indeed there are numerous CVD systematic reviews identifying over 110 different risk-scoring methods [29]. Patients are increasingly seeking guidance from online calculators to educate themselves and adjust for their personal lifestyle and habits. However, it is widely recognized that there is a lack of consistency among calculators. Small variations in risk estimates among calculators are to be expected, but as calculated risk estimate variation increases, calculators begin to categorize the same patient into different risk categories, with potentially life-threatening consequences [30]. The QRISK2 score is a prediction tool developed for the UK population because studies showed that the FRS overestimated CAD risk by 50−100% in white Europeans. It estimates the risk of a person developing CVD (stroke, transient ischemic attack, MI, angina) over the next 10 years. QRISK2 shows AUC statistics of 0.65 (95% Confidence Interval (CI), 0.61–0.68) and 0.71 (95% CI, 0.68–0.74), for men and women, respectively [7]. Compared to the Framingham score [31], which had an AUC-ROC of 0.724 (95% CI 0.720–0.728), and QRISK2 with an AUC-ROC 0.65, our best performing model outperformed both across all cohorts with an average AUC-ROC of 0.794; although the difference in AUC is limited and not a guaranteed of greater clinical utility. Other models to predict cardiovascular risk in the UK Biobank have found similar AUCs as ours when using more complex machine learning pipelines [11] or a risk assessment restricted to clinically common variable [10]. However, beyond mere performance, we believe that the strength of this study revolves around the difference in risk profile between the various risk groups and sex and the ability for the risk model to compare expected change in risk with both lifestyle changes and improved management of secondary risk factors.

The ANN model also included a number of features worthy of noting that are not generally included in current Framingham risk score or QRISK2 risk calculation. In laboratory data for instance, high levels of liver enzymes and urea were particularly important features in high-risk and disease-positive females but not in the low-risk population, these findings may support the existence of a liver-heart axis in CVD pathogenesis. An increased propensity of falls and weight loss, though unsurprising in the later cachectic stages of coronary disease and HF, are ranked highly in disease-positive participants only. This may further confirm these factors as a harbinger of poorer prognosis in this subset of patients. The model also identified being retired as an important feature in low-risk males, perhaps indicating a protective effect of vocation in this subgroup, but also may reflect the effect of age. This was true across the three risk strata, and in both males and females.

Of note, the model has identified ease of getting up in the morning as an important feature which warrants highlighting in our discussion. It has long been widely accepted that physical activity is protective against heart disease due to its various positive pleotropic and anti-inflammatory effects on coronaries. Exercise also plays a vital role in protecting arterial vasculature from the harm of long-standing hypertension as well as promotion of ventricular remodeling. The emergence of this feature likely reflects that patients who lead a sedentary lifestyle, and/or who may have a high BMI, and who have difficulty with initiating exercise, are at high risk of CAD and HF development. Further, the progression of CAD is slowed by increasing exercise tolerance and stamina on graded exercise and rehabilitation programs.

A history of cancer was a particularly important variable in the disease-positive cohorts, as was the number of operations reported on the intake questionnaire by males. The use of certain medications plays a predictive role in the female population of this study. Females taking endocrinologic medications were at higher risk of CAD-related outcomes, which may be due to a concomitant administration of diabetic medications for patients with high burden of vasculopathies and metabolic syndrome [32]. From a dietary perspective, salt and red meat intake were highly ranked as predictive features. This is consistent with large cohort studies [33,34]. Our model also served an important confirmatory role in other risk factors for CVD that are well-known and already included in many of these risk scoring systems. These included cholesterol levels, poor diabetic control, years of hypertension, smoking, and being on medications indicative of coronary vascular disease.

The model also played a novel risk factor discovery role on an agnostic level for CAD and HF. These include a slow walking pace, laboratory values of interest such as liver function tests and absolute neutrophil count, a positive history of cancer, and a high number of previous surgeries. These features can be easily incorporated into screening questionnaires for CAD and HF development, in particular online tools that can be available to populations without direct clinical care or laboratory resources. This study paves the way to providing personalized and accurate advice to patients. The biological plausibility of some features does require further study for validation before inclusion into future calculators, but they are important in that they emphasize the additive role of ML-methods as vital hypothesis-generating tools.

Our study investigated the efficacy of 6 different ML methods before one was selected as the strongest performer. We believe this to be pivotal to ensure high risk prediction performance metrics in ML. To the best of our knowledge, no other studies have screened several ML methods on a dataset as large as the UKB encompassing over 485,000 participants over a 7-year span [10,35]. Our study also uses a large variety of performance metrics to evaluate our model, making accurate comparisons possible against other ML methods internally, and externally against future models or currently used ones.

Another unique feature of our study is the division of participants into low-risk, high-risk and disease-positive patients at baseline. This led to a ranking of features more specific to each cohort and cohort-specific calibration, allowing for a highly tailored assessment of risk. Patients in reality are clinically and phenotypically different than one another, often to a large degree, which has complex interactions with the development of heterogenous diseases like coronary disease and heart failure. A screening tool for the development and progression of CAD or HF should likewise be different in each subset of the population depending on background and baseline risk. Our approach minimizes ranking features seemingly applicable to disease-free patients when in fact participants are symptomatic but undiagnosed at baseline, a significant limitation of other studies of similar objective or scope [11]. Furthermore, this study is the first to evaluate the entire depth and breadth of features available from the UK Biobank data repository, spanning clinical, laboratory and lifestyle realms. Previous studies did not have access to data such as triglycerides, markers of inflammation, and natriuretic peptides, or include genetic SNPs in their analysis; all of which are known to have bearing on CVD risk [11,36–39].

The choice to prioritize the minority class when adjusting class weights has greatly improved critical metrics like sensitivity and specificity. Two sets of model runs were performed: one without adjusting for minority class weight and the other with both over- and under-sampling strategies used. Both of these methods, meanwhile, failed to produce greater and more balanced prediction accuracy.

The capacity to penalize prediction mistakes within data samples linked to the minority class is the mechanism behind class weight adjustment. The training procedure emphasizes these data samples more by giving the minority class a higher weight. In the end, however, the final models do show excellent specificity at the expense of sensitivity. The clinical use case for this model favors focusing on specificity over sensitivity. For low- and high-risk groups, the consequence of a positive test is to intensify surveillance and management of risk factors, neither have a downside. On the other hand, there is a penalty for false negative as a false sense of being at low-risk and might not be as attentive as they could have been. This is why in this case, we opted to favor specificity over sensitivity.

This study should be viewed in the light of some limitations. First at the time of data harvest, the presence of disease was available only from hospital inpatient data and mortality records. Thus, patients who would have developed HF or CAD but who had not been hospitalized would not have been identified and the participants identified as having positive outcomes likely represent the higher end of the severity range. Inclusion of outpatient data may have added a significant number of events to the analysis, which may have helped to increase the model’s sensitivity. Second, while we believe that there is a high clinical relevance to use a prediction horizon of 7 year for the development of, or worsening of heart disease, it also limited the potential accuracy of our prediction models given the high degree of stochasticity in cardiovascular events. Finally, the UK Biobank cohort is ethnically homogeneous with over 94% of participants being white, although based on the substantial enrollment, there is still >25,000 non-white patients in the cohort.

In conclusion, in this study, we developed various ML-based algorithms to predict the development of or worsening of CVD both in people already affected by CVD and in the general population. As expected, the prediction models showed limited ability to predict events at the individual-patient level given the prediction horizon and the high degree of randomness in the timing of CVD-related events. However, the very high degree of accuracy of model calibration across the entire risk spectrum makes these algorithms useful both in terms of risk-based counselling and the implementation of prevention measures. Future endeavours should include the conversion of these algorithms in a patient-facing application with an interactive component that would display simulations of the expected change in risk of CVD associated with lifestyle changes and thromboprophylaxis strategies. Such a tool could be invaluable in individualizing risk management in people at risk of heart disease.

## Supporting information

S1 Methods Description of alternative approaches to create prediction models. (PDF)

S1 Table List of predictive features used for prediction models. (PDF)

S2 Table Single nucleotide polymorphism (SNP) included as potential features in predictive models for development or progression to heart failure and coronary artery disease. (PDF)

## References

[1] GBD 2017 Causes of Death Collaborators. Global, regional, and national age-sex-specific mortality for 282 causes of death in 195 countries and territories, 1980-2017: a systematic analysis for the Global Burden of Disease Study 2017. Lancet. 2018;392(10159):1736–88. doi: 10.1016/S0140-6736(18)32203-7 30496103 PMC6227606

[2] Kaptoge S, Pennells L, De Bacquer D, Cooney MT, Kavousi M, Stevens G, et al. World Health Organization cardiovascular disease risk charts: revised models to estimate risk in 21 global regions. The Lancet Global Health. 2019;7(10):e1332–45. doi: 10.1016/s2214-109x(19)30318-3 PMC7025029 31488387

[3] Arnett DK, Blumenthal RS, Albert MA, Buroker AB, Goldberger ZD, Hahn EJ, et al. 2019 ACC/AHA Guideline on the Primary Prevention of Cardiovascular Disease: A Report of the American College of Cardiology/American Heart Association Task Force on Clinical Practice Guidelines. Circulation. 2019;140(11):e596–646. doi: 10.1161/CIR.0000000000000678 30879355 PMC7734661

[4] Lloyd-Jones D, Adams RJ, Brown TM, Carnethon M, Dai S, De Simone G, et al. Executive summary: heart disease and stroke statistics--2010 update: a report from the American Heart Association. Circulation. 2010;121(7):948–54. doi: 10.1161/CIRCULATIONAHA.109.192666 20177011

[5] Greenland P, Alpert JS, Beller GA, Benjamin EJ, Budoff MJ, Fayad ZA, et al. 2010 ACCF/AHA guideline for assessment of cardiovascular risk in asymptomatic adults: a report of the American College of Cardiology Foundation/American Heart Association Task Force on Practice Guidelines. Circulation. 2010;122(25):e584–636. doi: 10.1161/CIR.0b013e3182051b4c 21098428

[6] Anderson TJ, Grégoire J, Pearson GJ, Barry AR, Couture P, Dawes M, et al. 2016 Canadian Cardiovascular Society Guidelines for the Management of Dyslipidemia for the Prevention of Cardiovascular Disease in the Adult. Can J Cardiol. 2016;32(11):1263–82. doi: 10.1016/j.cjca.2016.07.510 27712954

[7] Hippisley-Cox J, Coupland C, Vinogradova Y, Robson J, May M, Brindle P. Derivation and validation of QRISK, a new cardiovascular disease risk score for the United Kingdom: prospective open cohort study. BMJ. 2007;335(7611):136. doi: 10.1136/bmj.39261.471806.55 17615182 PMC1925200

[8] Berry JD, Lloyd-Jones DM, Garside DB, Greenland P. Framingham risk score and prediction of coronary heart disease death in young men. Am Heart J. 2007;154(1):80–6. doi: 10.1016/j.ahj.2007.03.042 17584558 PMC2279177

[9] Michos ED, Nasir K, Braunstein JB, Rumberger JA, Budoff MJ, Post WS, et al. Framingham risk equation underestimates subclinical atherosclerosis risk in asymptomatic women. Atherosclerosis. 2006;184(1):201–6. doi: 10.1016/j.atherosclerosis.2005.04.004 15907856

[10] Weng SF, Reps J, Kai J, Garibaldi JM, Qureshi N. Can machine-learning improve cardiovascular risk prediction using routine clinical data? PLoS One. 2017;12(4):e0174944. doi: 10.1371/journal.pone.0174944 28376093 PMC5380334

[11] Alaa AM, Bolton T, Di Angelantonio E, Rudd JHF, van der Schaar M. Cardiovascular disease risk prediction using automated machine learning: A prospective study of 423,604 UK Biobank participants. PLoS One. 2019;14(5):e0213653. doi: 10.1371/journal.pone.0213653 31091238 PMC6519796

[12] Dinh A, Miertschin S, Young A, Mohanty SD. A data-driven approach to predicting diabetes and cardiovascular disease with machine learning. BMC Med Inform Decis Mak. 2019;19(1):211. doi: 10.1186/s12911-019-0918-5 31694707 PMC6836338

[13] Quesada JA, Lopez‐Pineda A, Gil‐Guillén VF, Durazo‐Arvizu R, Orozco‐Beltrán D, López-Domenech A, et al. Machine learning to predict cardiovascular risk. Int J Clin Pract. 2019;73(10). doi: 10.1111/ijcp.13389 31264310

[14] Bycroft C, Freeman C, Petkova D, Band G, Elliott LT, Sharp K, et al. The UK Biobank resource with deep phenotyping and genomic data. Nature. 2018;562(7726):203–9. doi: 10.1038/s41586-018-0579-z 30305743 PMC6786975

[15] Useful materials - Access to the UK Biobank Resource. 2020. http://www.ukbiobank.ac.uk/key-documents/

[16] Sudlow C, Gallacher J, Allen N, Beral V, Burton P, Danesh J, et al. UK biobank: an open access resource for identifying the causes of a wide range of complex diseases of middle and old age. PLoS Med. 2015;12(3):e1001779. doi: 10.1371/journal.pmed.1001779 25826379 PMC4380465

[17] Van Hout CV, Tachmazidou I, Backman JD, Hoffman JD, Liu D, Pandey AK, et al. Exome sequencing and characterization of 49,960 individuals in the UK Biobank. Nature. 2020;586(7831):749–56. doi: 10.1038/s41586-020-2853-0 33087929 PMC7759458

[18] Lee H-Y, Chung W-J, Jeon H-K, Seo H-S, Choi D-J, Jeon E-S, et al. Impact of the β-1 adrenergic receptor polymorphism on tolerability and efficacy of bisoprolol therapy in Korean heart failure patients: association between β adrenergic receptor polymorphism and bisoprolol therapy in heart failure (ABBA) study. Korean J Intern Med. 2016;31(2):277–87. doi: 10.3904/kjim.2015.043 26879662 PMC4773723

[19] Morrison AC, Felix JF, Cupples LA, Glazer NL, Loehr LR, Dehghan A, et al. Genomic variation associated with mortality among adults of European and African ancestry with heart failure: the cohorts for heart and aging research in genomic epidemiology consortium. Circ Cardiovasc Genet. 2010;3(3):248–55. doi: 10.1161/CIRCGENETICS.109.895995 20400778 PMC3033765

[20] Vasan RS, Glazer NL, Felix JF, Lieb W, Wild PS, Felix SB, et al. Genetic variants associated with cardiac structure and function: a meta-analysis and replication of genome-wide association data. JAMA. 2009;302(2):168–78. doi: 10.1001/jama.2009.978-a 19584346 PMC2975567

[21] Villard E, Perret C, Gary F, Proust C, Dilanian G, Hengstenberg C, et al. A genome-wide association study identifies two loci associated with heart failure due to dilated cardiomyopathy. Eur Heart J. 2011;32(9):1065–76. doi: 10.1093/eurheartj/ehr105 21459883 PMC3086901

[22] Cappola TP, Li M, He J, Ky B, Gilmore J, Qu L, et al. Common variants in HSPB7 and FRMD4B associated with advanced heart failure. Circ Cardiovasc Genet. 2010;3(2):147–54. doi: 10.1161/CIRCGENETICS.109.898395 20124441 PMC2957840

[23] Lillvis JH, Lanfear DE. Progress toward genetic tailoring of heart failure therapy. Curr Opin Mol Ther. 2010;12(3):294–304. 20521218 PMC3048822

[24] Garnier S, Hengstenberg C, Lamblin N, Dubourg O, De Groote P, Fauchier L, et al. Involvement of BAG3 and HSPB7 loci in various etiologies of systolic heart failure: Results of a European collaboration assembling more than 2000 patients. Int J Cardiol. 2015;189:105–7. doi: 10.1016/j.ijcard.2015.04.003 25889438

[25] Cappola TP, Matkovich SJ, Wang W, van Booven D, Li M, Wang X, et al. Loss-of-function DNA sequence variant in the CLCNKA chloride channel implicates the cardio-renal axis in interindividual heart failure risk variation. Proc Natl Acad Sci U S A. 2011;108(6):2456–61. doi: 10.1073/pnas.1017494108 21248228 PMC3038744

[26] UK Biobank: Protocol for a large-scale prospective epidemiological resource. Protocol No: UKBB-PROT-09-06 (Main Phase). UK Biobank Coordinating Centre 21 March 2007. 2007.

[27] Endelman JB. Ridge regression and other kernels for genomic selection with R Package rrBLUP. The Plant Genome. 2011;4(3):250–5. doi: 10.3835/plantgenome2011.08.0024

[28] Brindle P, Beswick A, Fahey T, Ebrahim S. Accuracy and impact of risk assessment in the primary prevention of cardiovascular disease: a systematic review. Heart. 2006;92(12):1752–9. doi: 10.1136/hrt.2006.087932 16621883 PMC1861278

[29] Breswick A, Brindle P, Fahey T, Ebrahim S. Appendix K: A systematic review of risk scoring methods and clinical decision aids used in the primary prevention of coronary heart disease. London, UK: National Collaborating Centre for Primary Care and Royal College of General Practitioners; 2008. 21834196

[30] Allan GM, Nouri F, Korownyk C, Kolber MR, Vandermeer B, McCormack J. Agreement among cardiovascular disease risk calculators. Circulation. 2013;127(19):1948–56. doi: 10.1161/CIRCULATIONAHA.112.000412 23575355

[31] Wilson PW, D’Agostino RB, Levy D, Belanger AM, Silbershatz H, Kannel WB. Prediction of coronary heart disease using risk factor categories. Circulation. 1998;97(18):1837–47. doi: 10.1161/01.cir.97.18.1837 9603539

[32] Eckel RH, Grundy SM, Zimmet PZ. The metabolic syndrome. Lancet. 2005;365(9468):1415–28. doi: 10.1016/S0140-6736(05)66378-7 15836891

[33] Du H, Li L, Bennett D, Guo Y, Key TJ, Bian Z, et al. Fresh fruit consumption and major cardiovascular disease in China. N Engl J Med. 2016;374(14):1332–43. doi: 10.1056/NEJMoa1501451 27050205 PMC4896382

[34] de Oliveira Otto MC, Mozaffarian D, Kromhout D, Bertoni AG, Sibley CT, Jacobs DR Jr, et al. Dietary intake of saturated fat by food source and incident cardiovascular disease: the Multi-Ethnic Study of Atherosclerosis. Am J Clin Nutr. 2012;96(2):397–404. doi: 10.3945/ajcn.112.037770 22760560 PMC3396447

[35] Ambale-Venkatesh B, Yang X, Wu CO, Liu K, Hundley WG, McClelland R, et al. Cardiovascular Event Prediction by Machine Learning: The Multi-Ethnic Study of Atherosclerosis. Circ Res. 2017;121(9):1092–101. doi: 10.1161/CIRCRESAHA.117.311312 28794054 PMC5640485

[36] Assmann G, Cullen P, Schulte H. Simple scoring scheme for calculating the risk of acute coronary events based on the 10-year follow-up of the prospective cardiovascular Münster (PROCAM) study. Circulation. 2002;105(3):310–5. doi: 10.1161/hc0302.102575 11804985

[37] Emerging Risk Factors Collaboration, Kaptoge S, Di Angelantonio E, Pennells L, Wood AM, White IR, et al. C-reactive protein, fibrinogen, and cardiovascular disease prediction. N Engl J Med. 2012;367(14):1310–20. doi: 10.1056/NEJMoa1107477 23034020 PMC3714101

[38] Natriuretic Peptides Studies Collaboration, Willeit P, Kaptoge S, Welsh P, Butterworth A, Chowdhury R, et al. Natriuretic peptides and integrated risk assessment for cardiovascular disease: an individual-participant-data meta-analysis. Lancet Diabetes Endocrinol. 2016;4(10):840–9. doi: 10.1016/S2213-8587(16)30196-6 27599814 PMC5035346

[39] Burghardt TP. Demographic model for inheritable cardiac disease. Arch Biochem Biophys. 2019;672:108056. doi: 10.1016/j.abb.2019.07.021 31356777

