# Supplementary material for: Predicting the future risk and outcomes of severe heart failure and coronary artery disease with machine learning in the UK Biobank Cohort
Source: PLoS One. 2025 Sep 10;20(9):e0329461. doi: 10.1371/journal.pone.0329461 (PMC12422514; doi:10.1371/journal.pone.0329461)
Supplement: S1 Methods — (PDF) [file pone.0329461.s001.pdf]

S1 Methods.

Various predictive models were compared in this study. The objective was to balance model performance with the refinement of risk profiles in such a way that it reflects real-world patient scenarios. Therefore, all candidate predictors were used in model training across each of the evaluated approaches. While models were permitted to examine the influence of individual variables, no predictors were excluded entirely. This strategy allowed for consistent comparison of risk factors across various data stratifications. The following models were compared in the analysis:.: bootstrap logistic regression (LR) with stepwise feature selection, Lasso LR, Support Vector Machines (SVM), Random Forest (RF), Gradient Boosting Machines (GBM), Deep Neural Network (DNN). Relevant details for each method are provided below:

- Lasso Logistic Regression (LR)[1]: this method works by finding a linear relation between one or more of patients' covariates and a binary outcome of interest. In this study, LR method was used with a Lasso regularization function.

- Bootstrap logistic regression (LR) with feed forward feature selection: the method runs for n = 50 bootstraps. For each bootstrap, part of the training data (70%) was randomly selected and LR with feed forward feature selection was used to find statistically important features. At the end of 50 bootstraps unique feature sets were chosen from all bootstrap runs. The selected feature set was then used for subsequent analysis which is to tune for the best parameter and test on an unseen set.

- Support Vector Machines (SVM)[2]: This method tries to find a separation plane between classes of data in such a way that the separating plane has the largest distance to its nearest training data of each class (the so-called maximum margin). Since in most cases, all data points are not entirely located on each side of the separating hyperplane, SVM implementations allow points to be misclassified (being placed on the wrong side of the hyperplane) at a certain cost value (known as soft margin). SVM is relatively robust to noisy data and are easy to interpret. For this study, we used the linear kernel to identify a separating plane between two classes of data.

- Random Forest (RF)[3]: constructs a multitude decision tree and performs a majority voting on the decisions made through all the constructed trees. The decision tree works by passing data through a multi-level tree like structure in which at each level a yes/no question is asked about the data. The answer to each question at each level eventually leads us to a predicted class. When training from data, each tree within the random forest randomly selects a subset of patients and features to learn from. The idea here is to have multiple passes on the data (through each decision tree) in such a way that the overall variance of the model is minimized. For this method, number and maximum depth of trees were optimized through the cross-validation scheme.

- Gradient Boosting Machines (GBM)[4]: The idea behind boosting methods is to start by creating an initial model from the data and start improving it in a gradual, additive and sequential manner by focusing on those data that the previous models did not accurately predict. The combination of best possible model with the previous models is expected to perform better. For this study, the learning model works based on the decision trees technique. Therefore, number and maximum depth of trees were optimized through the cross-validation scheme.

- Artificial Neural Network (ANN)[5]: a learning model that consists of highly interconnected processing elements across various layers which process the information that pass through them to find a mapping between the input information and the output results. For this study, a four-layer ANN was used with n = number of features in the dataset for the first layer and twice nodes as in the first layer for second and third layers. Each layer follows with a dropout (with a rate of 0.5) and batch normalization layers. The last layer of the network has sigmoid activation function to convert the output of the previous

layer to probability values. We used a linear kernel activation and HE uniform kernel initializer. Adam optimizer[6] was used to optimize the network's weights with a learning rate of 0.01. The choice of the specific network architecture used in this study was first derived from similar publications and the specifics about the number of nodes and hidden layers was confirmed by comparing the performance metrics of a randomly selected subset of data for at-risk patients. Other parameters such as drop out amount, kernel activation, kernel initializer and optimizer was tuned using a random search.
