# Supplementary material for: Predicting the future risk and outcomes of severe heart failure and coronary artery disease with machine learning in the UK Biobank Cohort
Source: PLoS One. 2025 Sep 10;20(9):e0329461. doi: 10.1371/journal.pone.0329461 (PMC12422514; doi:10.1371/journal.pone.0329461)
Supplement: S1 Table — (PDF) [file pone.0329461.s002.pdf]

**S1 Table.** List of predictive features used for prediction models

| **Major risk domain** | **Specific predictive features** |
|---|---|
| Self-reported physical activity, screen time and sleep duration/quality | - Daily duration of moderate and vigorous physical activity<br>- Daily walking distance, usual walking pace<br>- Frequency of stairs climbing<br>- Frequency of walking/standing, manual/heavy physical labour at work<br>- Daily screen time (computer and television)<br>- Work schedule in shift<br>- Sleep quality (duration, insomnia, napping, snoring)<br>- Usual chronotype, ease of getting up in the morning |
| Genetics | - Single nucleotide polymorphism (supplementary Table 2)<br>- Selected medical history in 1st degree relatives:<br>  - Cardiovascular disease (note 1)<br>  - Cancer (all types)<br>  - Severe lung/respiratory disease<br>  - Severe neurological/mental disease |
| Medical examination, laboratory investigations and medications | - Age at recruitment, self-rating of overall health<br>- Body measurements (weight, height, body mass index)<br>- Recent wheeze or whistling in chest, blood pressure and pulse rate<br>- Laboratory investigations:<br>  - Complete blood count with white blood cell differential<br>  - Lipid and glycemic profile<br>  - Liver and renal function profile<br>  - Sex hormones<br>  - Vitamin D<br>- Medications (grouped by class of agents)<br>  - Cardiovascular and antithrombotics (note 2)<br>  - Metabolic, musculoskeletal, hormonal<br>  - Analgesic, psychoactive agents, anti-asthmatic medication<br>  - Vitamin and nutritional supplements |
| Previous medical history | - Recent changes in diet or weight<br>- Recent falls and fractures<br>- Body size at age 10 years, breastfeeding history<br>- Previous surgical operations, cancer history<br>- History of major previous medical conditions (grouped by categories)<br>- Lifetime duration of asthma, diabetes, allergic diseases<br>- Lifetime duration of elevated blood pressure |
| Nutrition | - Usual poultry, red meat (beef, lamb, pork), processed meat intake (frequency)<br>- Usual fish (oily and non-oily) intake (frequency)<br>- Usual cereal intake (type and frequency)<br>- Usual milk/cheese/butter intake (type and frequency)<br>- Usual fruit and vegetable intake (number of portions per day)<br>- Salt intake (frequency)<br>- Tea, water, coffee intake (amount per day)<br>- Usual temperature of consumption for hot drinks |
| Socioeconomic status, alcohol and tobacco use | - Alcohol intake frequency<br>- Tobacco intake frequency, lifetime exposure<br>- Education, employment status, income and Townsend deprivation index<br>- Immigration status, ethnic background<br>- Household composition and stability |

Note 1: Includes cardiovascular disease, strokes, diabetes, and high blood pressure
Note 2: Only in participants with existing cardiovascular disease at enrollment
