# Supplementary material for: Predicting the future risk and outcomes of severe heart failure and coronary artery disease with machine learning in the UK Biobank Cohort
Source: PLoS One. 2025 Sep 10;20(9):e0329461. doi: 10.1371/journal.pone.0329461 (PMC12422514; doi:10.1371/journal.pone.0329461)
Supplement: S2 Table — (PDF) [file pone.0329461.s003.pdf]

S2 Table. Single nucleotide polymorphism (SNP) included as potential features in predictive models for development or progression to heart failure and coronary artery disease.

**Gene**	**SNPs**	**Protein**	**Putative function**	**Refs**
ADAMTS12	3	ADAM metallopeptidase with TS12	Modulation of normal neutrophil apoptosis	7, 8
ADRB1	1	Adrenoceptor beta 1	Encodes Beta 1 adrenergic receptor	9, 10
ADRB2	3	Adrenoceptor beta 2	Encodes Beta 2 adrenergic receptor	9, 11
BAG3	2	BAG cochaperone 3	Targets aggregation-prone proteins to autophagic degradation	11-14
CEP85L	6	Centrosomal protein 85 like	protein encoded by this gene was identified as a breast cancer antigen	15, 16
CLCNKA	5	Chloride voltage-gated channel Ka	encoded protein thought to function in salt reabsorption in the kidney and potassium recycling in the inner ear	17, 18
CMTM7	1	CKLF like MARVEL transmembrane domain containing 7	This gene acts as a tumor suppressor that regulates G1/S transition in the cell cycle, and epidermal growth factor receptor/protein kinase B signaling during tumor pathogenesis	7, 19
FRMD4B	6	FERM domain containing 4B	GRP1-binding protein which contains a FERM protein interaction domain as well as two coiled coil domains. This protein may play a role as a scaffolding protein	20, 21
GRID1	3	Glutamate ionotropic receptor delta type subunit 1	Receptor for glutamate. L-glutamate acts as an excitatory neurotransmitter at many synapses in the central nervous system.	15, 22
GRK5	2	G protein-coupled receptor kinase 5	Serine/threonine kinase that phosphorylates preferentially the activated forms of a variety of G-protein-coupled receptors (GPCRs). Initiates beta-arrestin-mediated receptor desensitization, internalization, and signaling events leading to their down-regulation.	11, 23
IFRD1	1	Interferon related developmental regulator 1	Unclear role. Could play a role in regulating gene activity in the proliferative and/or differentiative pathways induced by NGF. May be an autocrine factor that attenuates or amplifies the initial ligand-induced signal.	7, 22
LRIG3	1	Leucine rich repeats and immunoglobulin like domains 3	May play a role in craniofacial and inner ear morphogenesis during embryonic development	24
MAF	1	MAF bZIP transcription factor	The protein encoded by this gene is a DNA-binding, leucine zipper-containing transcription factor that acts as a homodimer or as a heterodimer. This protein plays a role in the regulation of several cellular processes, including embryonic lens fiber cell development, increased T-cell susceptibility to apoptosis, and chondrocyte terminal differentiation.	25
NOS3	2	Nitric oxide synthase 3	Produces nitric oxide (NO) which is implicated in vascular smooth muscle relaxation through a cGMP-mediated signal transduction pathway.	26

OTUD7A	1	OTU deubiquitinase 7A	Has deubiquitinating activity towards 'Lys-11'-linked polyubiquitin chains.	7, 27
**Gene**	**SNPs**	**Protein**	**Putative function**	**Refs**
PARVA	1	Parvin alpha	Encoded protein plays a role in sarcomere organization and in smooth muscle cell contraction.	7, 28
PCSK6	4	Proprotein convertase subtilisin/kexin type 6	Serine endoprotease that processes various proproteins by cleavage at paired basic amino acids. Likely functions in the constitutive secretory pathway, with unique restricted distribution in both neuroendocrine and non-neuroendocrine tissues.	20, 29
SLC35F1	1	Solute carrier family 35 member F1	Involved in the maintenance of mitochondrial membrane potential in pancreatic ductal adenocarcinoma (PDAC) cells. Promotes pancreatic ductal adenocarcinoma (PDAC) cell growth.	15, 30
TMEM232	6	Transmembrane protein 232	Membrane protein with unknown function	22
WWOX	6	WW domain containing oxidoreductase	Putative oxidoreductase. Acts as a tumor suppressor and plays a role in apoptosis. Required for normal bone development	15, 31
N/A	1	Uncharacterized LOC105372932 (rs12733856)	N/A	7
N/A	1	NCR chr14:90213566 (GRCh38.p12) (rs4528684)	N/A	7
N/A	1	NCR chr10:77684995 (GRCh38.p12) (rs4979906)	N/A	7
N/A	1	NCR chr12:131378358 (GRCh38.p12) (rs7965445)	N/A	7
N/A	1	NCR (GRCh38.p12) (rs8017423)	N/A	7

REFERENCES

1. McCullagh P and Nelder J, Generlized Linear Models. 2nd edition ed. CRC Monographs on Statistics and Applied Probability. 1989: Chapman & Hall. 532.
2. Cortes C and Vapnik V. Support-vector networks. Machine Learning, 1995 20:273-297.
3. Ho T. Random decision forests. in Proceedings of 3rd International Conference on Document Analysis and Recognition. 1995. Montreal, Canada: IEEE.
4. Friedman J. Greedy function approximation: A gradiet boosting machine. The Annals of Statistics, 2001 29(4):1189-1232.
5. Schmidhuber J. Learning Complex, Extended Sequences Using the Principle of History Compression. Neural Computation, 1992 4(2):234-242.
6. Kingma D and Ba J. Adam: A method for stochastic optimization. in 3rd International Conference for Learning Representations. 2015. San Diego.
7. Morrison AC, Felix JF, Cupples LA, et al. Genomic variation associated with mortality among adults of European and African ancestry with heart failure: the cohorts for heart and aging research in genomic epidemiology consortium. Circ Cardiovasc Genet, 2010 3(3):248-55.
8. Moncada-Pazos A, Obaya AJ, Llamazares M, et al. ADAMTS-12 Metalloprotease Is Necessary for Normal Inflammatory Response. Journal of Biological Chemistry, 2012 287(47):39554-39563.
9. Lee HY, Chung WJ, Jeon HK, et al. Impact of the beta-1 adrenergic receptor polymorphism on tolerability and efficacy of bisoprolol therapy in Korean heart failure patients: association between beta adrenergic receptor polymorphism and bisoprolol therapy in heart failure (ABBA) study. Korean J Intern Med, 2016 31(2):277-87.
10. Miao Y, Chen H, and Li M. MiR-19a overexpression contributes to heart failure through targeting ADRB1. International Journal of Clinical and Experimental Medicine, 2015 8(1):642-649.
11. Lillvis JH and Lanfear DE. Progress toward genetic tailoring of heart failure therapy. Curr Opin Mol Ther, 2010 12(3):294-304.

12. Villard E, Perret C, Gary F, et al. A genome-wide association study identifies two loci associated with heart failure due to dilated cardiomyopathy. Eur Heart J, 2011 32(9):1065-76.
13. Garnier S, Hengstenberg C, Lamblin N, et al. Involvement of BAG3 and HSPB7 loci in various etiologies of systolic heart failure: Results of a European collaboration assembling more than 2000 patients. Int J Cardiol, 2015 189:105-7.
14. Sturner E and Behl C. The Role of the Multifunctional BAG3 Protein in Cellular Protein Quality Control and in Disease. Front Mol Neurosci, 2017 10(177):177.
15. Vasan RS, Glazer NL, Felix JF, et al. Genetic variants associated with cardiac structure and function: a meta-analysis and replication of genome-wide association data. JAMA, 2009 302(2):168-78.
16. Mungall AJ, Palmer SA, Sims SK, et al. The DNA sequence and analysis of human chromosome 6. Nature, 2003 425(6960):805-11.
17. Cappola TP, Matkovich SJ, Wang W, et al. Loss-of-function DNA sequence variant in the CLCNKA chloride channel implicates the cardio-renal axis in interindividual heart failure risk variation. Proc Natl Acad Sci U S A, 2011 108(6):2456-61.
18. Gerbino A, De Zio R, Russo D, et al. Role of PKC in the Regulation of the Human Kidney Chloride Channel ClC-Ka. Sci Rep, 2020 10(1):10268.
19. Huang ZM, Li PL, Yang P, et al. Overexpression of CMTM7 inhibits cell growth and migration in liver cancer. Kaohsiung J Med Sci, 2019 35(6):332-340.
20. Cappola TP, Li M, He J, et al. Common variants in HSPB7 and FRMD4B associated with advanced heart failure. Circ Cardiovasc Genet, 2010 3(2):147-54.
21. Klarlund JK, Holik J, Chawla A, Park JG, Buxton J, and Czech MP. Signaling complexes of the FERM domain-containing protein GRSP1 bound to ARF exchange factor GRP1. J Biol Chem, 2001 276(43):40065-70.
22. Ota T, Suzuki Y, Nishikawa T, et al. Complete sequencing and characterization of 21,243 full-length human cDNAs. Nat Genet, 2004 36(1):40-5.
23. Kunapuli P and Benovic JL. Cloning and expression of GRK5: a member of the G protein-coupled receptor kinase family. Proc Natl Acad Sci U S A, 1993 90(12):5588-92.
24. Guo DS, Holmlund C, Henriksson R, and Hedman H. The LRIG gene family has three vertebrate paralogs widely expressed in human and mouse tissues and a homolog in Ascidiacea. Genomics, 2004 84(1):157-165.
25. Chakhtoura M, Fang M, Cubas R, et al. Germinal Center T follicular helper (GC-Tfh) cell impairment in chronic HIV infection involves c-Maf signaling. PLoS Pathog, 2021 17(7):e1009732.
26. Janssens SP, Shimouchi A, Quertermous T, Bloch DB, and Bloch KD. Cloning and expression of a cDNA encoding human endothelium-derived relaxing factor/nitric oxide synthase. J Biol Chem, 1992 267(21):14519-22.
27. Virdee S, Ye Y, Nguyen DP, Komander D, and Chin JW. Engineered diubiquitin synthesis reveals Lys29-isopeptide specificity of an OTU deubiquitinase. Nat Chem Biol, 2010 6(10):750-7.
28. Olski TM, Noegel AA, and Korenbaum E. Parvin, a 42 kDa focal adhesion protein, related to the alpha-actinin superfamily. J Cell Sci, 2001 114(Pt 3):525-38.
29. Kiefer MC, Tucker JE, Joh R, Landsberg KE, Saltman D, and Barr PJ. Identification of a second human subtilisin-like protease gene in the fes/fps region of chromosome 15. DNA Cell Biol, 1991 10(10):757-69.
30. Clark HF, Gurney AL, Abaya E, et al. The secreted protein discovery initiative (SPDI), a large-scale effort to identify novel human secreted and transmembrane proteins: a bioinformatics assessment. Genome Res, 2003 13(10):2265-70.
31. Bednarek AK, Laflin KJ, Daniel RL, Liao Q, Hawkins KA, and Aldaz CM. WWOX, a novel WW domain-containing protein mapping to human chromosome 16q23.3-24.1, a region frequently affected in breast cancer. Cancer Res, 2000 60(8):2140-5.
